# Real-World Effectiveness of Mineralocorticoid Receptor Antagonists in Primary Aldosteronism

**DOI:** 10.3389/fendo.2021.625457

**Published:** 2021-03-26

**Authors:** Yuta Tezuka, Adina F. Turcu

**Affiliations:** ^1^ Division of Metabolism, Endocrinology and Diabetes, University of Michigan, Ann Arbor, MI, United States; ^2^ Division of Nephrology, Endocrinology and Vascular Medicine, Tohoku University Graduate School of Medicine, Sendai, Japan

**Keywords:** primary aldosteronism, renin, mineralocorticoid receptor (MR) antagonist, adrenal disorders, hypertension, aldosterone, adrenal, adrenal cortex

## Abstract

**Objective:**

To investigate how often target renin is pursued and achieved in patients with primary aldosteronism (PA) and other low renin hypertension (LRH) treated with mineralocorticoid receptor antagonists (MRAs), as reversal of renin suppression was shown to circumvent the enhanced cardiovascular and renal morbidity and mortality in these patients.

**Patients and Methods:**

We conducted a retrospective cohort study of patients with PA and LRH treated with MRAs in an academic outpatient practice from January 1, 2000, through May 31, 2020.

**Results:**

Of 30,777 patients with hypertension treated with MRAs, only 7.3% were evaluated for PA. 163 patients (123 with PA) had renin followed after MRA initiation. After a median follow-up of 124 [interquartile range, 65-335] days, 70 patients (43%) no longer had renin suppression at the last visit. The proportion of those who achieved target renin was higher in LRH than in PA (53% vs. 40%). Lower baseline serum potassium, lower MRA doses, and beta-blocker use were independently associated with lower odds of achieving target renin in PA, while male sex was associated with target renin in LRH. Overall, 50 patients (30.7%) had 55 adverse events, all from spironolactone, and 26 patients (52%) were switched to eplerenone or had a spironolactone dose reduction.

**Conclusion:**

Despite evidence that reversal of renin suppression confers cardio-renal protection in patients with PA and LRH, renin targets are followed in very few and are achieved in under half of such patients seen in an academic setting, with possibly even lower rates in community practices.

## Introduction

Primary aldosteronism (PA) is a common form of secondary hypertension, accounting for up to 20% of resistant hypertension cases (1–3). Compared to sex- and age- matched individuals with essential hypertension and equivalent blood pressure, patients with PA have a higher risk of cardiovascular and renal complications, including atrial fibrillation, coronary artery disease, strokes, renal insufficiency, and death (4, 5). Such complications are partly mediated by excessive mineralocorticoid receptor (MR) activation in target tissues, which promotes myocardial fibrosis, left ventricular hypertrophy, increased carotid intima-media thickness, endothelial dysfunction, and microalbuminuria (6–12). Mineralocorticoid receptor antagonists (MRAs) are the mainstay of medical treatment for PA (13). While small observational studies showed that MRAs can be efficacious for blood pressure control and cardiovascular protection even in low doses (14–16), large retrospective cohort studies of patients with PA and essential hypertension suggest that the cardio-renal benefits of MRA therapy in PA patients are maximized when renin is no longer suppressed (4, 17, 18).

The clinical benefits of MRA therapy on blood pressure control and cardiovascular morbidity and mortality has also been demonstrated more broadly in resistant hypertension (19–21). Moreover, low renin has been associated with cardiovascular risk in patients with “essential hypertension” (22–24) and it has been shown to be a predictor of blood pressure response to MRAs in this population (25). Therefore, abrogation of renin suppression is suggestive of therapeutic MR blockade that overcomes the excessive amounts of aldosterone or other MR activators.

In this study, conducted in a large academic clinical practice that includes both primary care and specialty services, we aimed to: 1) assess how often MRA therapy is titrated to target renin levels; and 2) identify factors that preclude adequate MRA dose titration to overcome renin suppression, such as side effects or concomitant medications that contribute to alterations of renin-angiotensin-aldosterone-system (RAAS).

## Patients and Methods

### Study Participants

**Figure 1:**
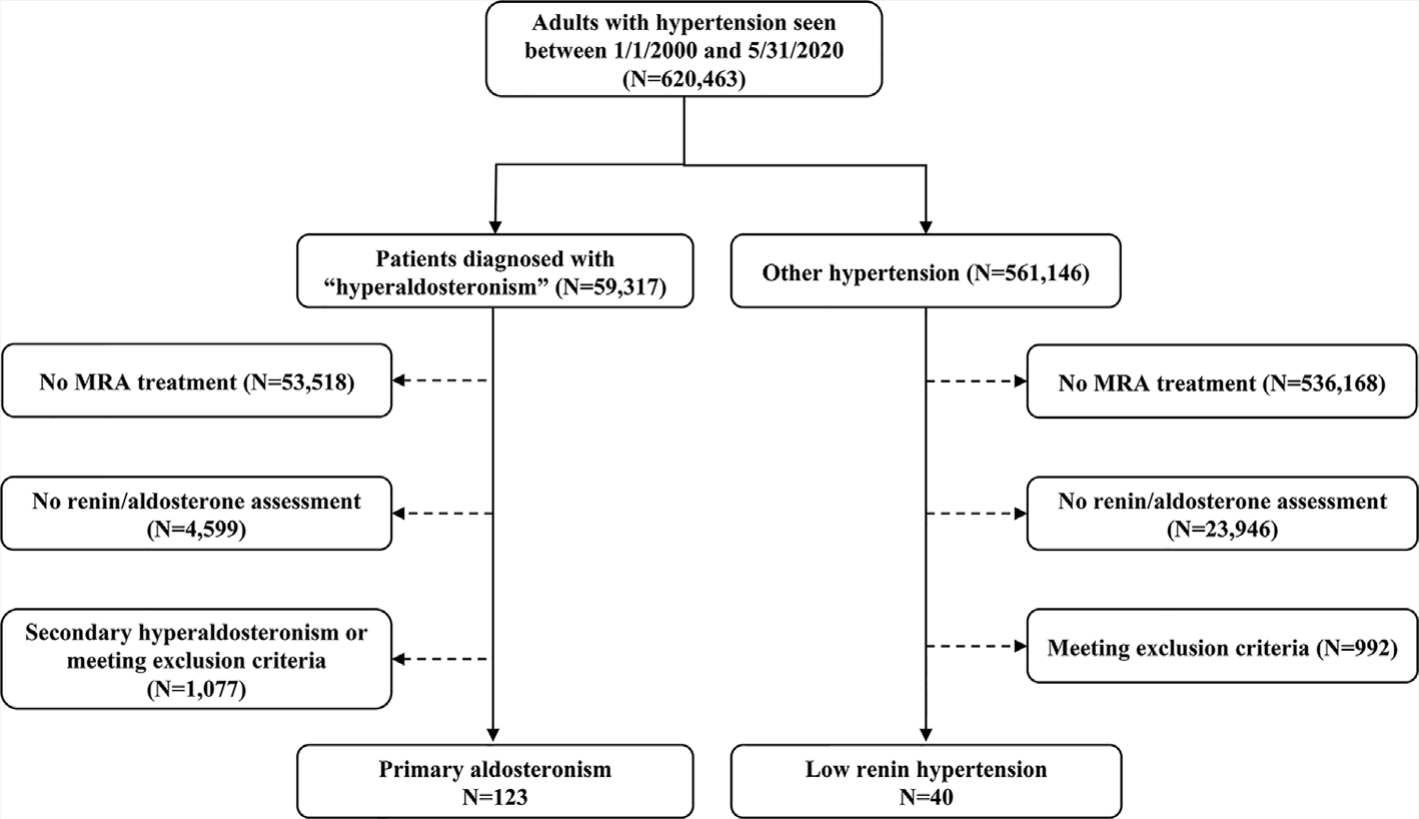
Selection of study participants. MRA, mineralocorticoid receptor antagonist; PA, primary aldosteronism.

We employed an internal database search engine, DataDirect (26), to identify patients with low-renin hypertension treated with MRA between January 1^st^, 2000 and June 1^st^, 2020 (**Figure 1**). We included patients with hypertension who had: 1) suppressed renin prior to initiation of MRA therapy, and 2) follow-up renin measurements after MRA initiation available in our medical records. We excluded patients with: end-stage renal disease; Cushing syndrome; glucocorticoid use; adrenal cortical carcinoma; congenital adrenal hyperplasia; and critically ill patients. We also excluded patients who did not have follow-up renin within 18 months after MRA initiation. This study was conducted with the University of Michigan Internal Review Boards approval (HUM00055821). A waiver of consent was granted for the retrospective review of medical records.

### Clinical Information

Data extracted from medical records included: demographics, body mass index (BMI), blood pressure, medical diagnoses, medications, serum electrolytes, the estimated glomerular filtration ratio (eGFR), plasma aldosterone concentration (PAC), plasma renin, both before and after MRA initiation, as well as side effects related to MRA use. To compile results, eplerenone doses were converted to equivalent spironolactone doses by dividing by 2 (18, 27, 28).

### Measurement of Renin and Definition of PA Diagnosis

Plasma renin activity (PRA) was available in the majority of patients (74.8%), and it was measured as previously reported (29). Direct renin concentration (DRC) was measured after February 2018 and it was the only test available in the remaining patients (25.2%). DRC was measured with a DiaSorin Liaison competitive chemiluminescent immunoassay with a coefficient of variability <10%. A conversion factor of 1:8 was used to transform DRC values into PRA equivalents following rigorous in-house analysis of both assays over a 9-month transition period. Renin was considered suppressed when PRA was <1.0 ng/mL/h (to convert to pmol/L, multiply by 0.0030) and DRC was <8.0 pg/mL (to convert to pmol/L, multiply by 0.0237), and renin values above these thresholds were set as target following MRA therapy. The diagnosis of PA was established based on one of the following criteria: an oral salt loading test followed by a 24-hour urinary aldosterone >12 μg (to convert to μmol, multiply by 0.0028); an intravenous saline infusion test followed by a PAC >6.8 ng/dL (to convert to nmol/L, multiply by 0.0277) at 4 hours; or PAC ≥20 ng/dL along with suppressed renin (13). The laterality of hyperaldosteronism was confirmed by adrenal venous sampling, as previously reported (30).

### Statistical Analysis

The Mann-Whitney *U* test was used for comparison of continuous variables between two independent groups. The paired *t*-test (for normal data distribution) or Wilcoxon signed-rank test (skewed data distribution) were used to compare continuous variables prior to and after MRA therapy within the same patients. The Chi-squared test and Fisher’s exact test were used for comparison of proportions between two groups. Linear regression analysis was used to assess relationships between continuous variables. Multiple logistic regression with backward stepwise selection was employed to evaluate predictors of target renin achievement following MRA treatment. All statistical analyses were performed with StatFlex software (version 7.0; Artech Co, Ltd, Osaka, Japan) or Prism (version 8.0; GraphPad Software, La Jolla, CA). Statistical significance was accepted at *p* values smaller than 0.05.

## Results

### Demographics and Clinical Characteristics of Study Participants

Of 620,463 adult patients with hypertension seen across our institution during the study period, 59,317 (10%) patients had a documented diagnosis of “hyperaldosteronism” (**Figure 1**). Only 5% of all patients and 9.8% of those with hyperaldosteronism were prescribed an MRA. In total, 123 patients with PA and 40 patients with other low-renin hypertension (LRH) met all inclusion criteria (**Figure 1**). The number of the patients meeting entry criteria increased progressively since 2013 (13 before 2010, 51 from 2011 through 2015, and 97 since 2016; **Supplemental Figure 1**). The median age of all participants at study entry was 56 (range, 19 to 84) years, and it was similar in both groups (**Table 1**). Patients with PA were more frequently men (60%), while those with LRH were more often women (67.5%, *p*=0.02). The median number of antihypertensive agents was 3 (interquartile range: 2 to 4) in both groups. Patients with LRH had a higher systolic blood pressure (159 [137, 182] vs.148 [132, 162] mmHg, *p*=0.03). Beta-blockers, RAAS inhibitors (including angiotensin converting enzyme inhibitors and angiotensin receptor blockers), and calcium channel blockers were prescribed in 64%, 53%, and 69% of the patients, respectively. Patients with PA were treated more often with calcium channel blockers (75% vs. 53%, *p*=0.008), and potassium supplements (45% vs. 20%, *p*=0.01) than those without PA (**Table 1**).

**Table 1:** Baseline demographics of study participants.

Variables	All	PA	LRH	*p*
N	163	123	40	
Age (years)	56 [46, 64]	56 [47, 64]	56 [46, 67]	0.99
Women (N, %)	76 (46.6%)	49 (39.8%)	27 (67.5%)	0.02
BMI (kg/m^2^)	32.9 [28.7, 36.5]	32.9 [29.0, 36.0]	33.3 [28.4, 37.1]	0.92
SBP (mmHg)	149 [134, 168]	148 [132, 162]	159 [137, 182]	0.03
DBP (mmHg)	84 [75, 92]	84 [75, 91]	85 [76, 99]	0.13
Serum potassium (mM)	3.8 [3.5, 4.2]	3.7 [3.5, 4.2]	4.0 [3.5, 4.2]	0.24
eGFR (mL/min/1.73m^2^)	81 [64, 94]	81 [65, 92]	82 [61, 96]	0.86
PAC (ng/dL)	18.8 [13.2, 26.5]	21.0 [16.3, 29.7]	11.2 [8.4, 16.6]	<0.001
PRA (ng/mL/hr) [N = 122]	0.3 [0.1, 0.6]	0.2 [0.1, 0.6]	0.4 [0.2, 0.7]	0.06
DRC (pg/mL) [N = 41]	2.1 [2.1, 3.5]	2.1 [2.1, 2.1]	2.3 [2.1, 4.3]	0.052
ARR (ng/dL per ng/mL/h)	61.0 [32.2, 124.4]	79.5 [38.6, 170.0]	28.9 [18.5, 47.1]	<0.001
Follow-up duration (days)	124 [65, 335]	175 [73, 342]	80 [35, 173]	0.008
MRA dosage at the last visit (mg/day)	50.0 [25.0, 50.0]	50.0 [25.0, 50.0]	25.0 [25.0, 50.0]	0.18
MRA-related side effects (N, %)	50 (30.7%)	41 (33.3%)	9 (22.5%)	0.20
Smoking history (N, %)	59 (37.1%)	43 (35.8%)	16 (41.0%)	0.56
Diabetes mellitus (N, %)	52 (31.9%)	35 (28.5%)	17 (42.5%)	0.10
Cardiovascular disease (N, %)	33 (20.2%)	23 (18.7%)	10 (25.0%)	0.39
Stroke (N, %)	16 (9.8%)	10 (8.1%)	6 (15.0%)	0.20
Antihypertensive agents (N)	3.0 [2.0, 4.0]	3.0 [2.0, 4.0]	3.0 [2.0, 4.0]	0.58
Alpha-blocker (N, %)	22 (13.5%)	18 (14.6%)	4 (10.0%)	0.46
Beta-blocker (N, %)	105 (64.4%)	80 (65.0%)	25 (62.5%)	0.77
Central agonists (N, %)	39 (23.9%)	26 (21.1%)	13 (32.5%)	0.14
Potassium-wasting diuretics (N, %)	69 (42.3%)	54 (43.9%)	15 (37.5%)	0.48
Potassium-sparing diuretics (N, %)	10 (6.1%)	9 (7.3%)	1 (2.5%)	0.27
RAAS inhibitors (N, %)	87 (53.4%)	61 (49.6%)	26 (65.0%)	0.09
Calcium channel blocker (N, %)	113 (69.3%)	92 (74.8%)	21 (52.5%)	0.008
Other antihypertensives (N, %)	9 (5.5%)	7 (5.7%)	2 (5.0%)	0.87
Potassium replacement (N, %)	63 (38.7%)	55 (44.7%)	8 (20.0%)	0.005

PA, primary aldosteronism; LRH, low renin essential hypertension; BMI, body mass index; SBP, systolic blood pressure; DBP, diastolic blood pressure; eGFR, estimated glomerular filtration ratio; PAC, plasma aldosterone concentration; PRA, plasma renin activity; DRC, direct renin concentration; ARR, aldosterone-to-renin ratio; RAAS, renin-angiotensin-aldosterone system; MRA, mineralocorticoid receptor antagonist. RAAS inhibitors include angiotensin-converting enzyme inhibitors and angiotensin receptor blockers. Continuous variables are shown as median [interquartile range].

### Rates of Target Renin Achievement After MRA Initiation

**Figure 2:**
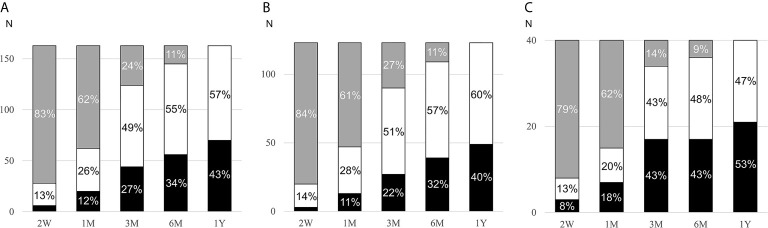
Cumulative rates of target renin achievement during MRA therapy. MRA, mineralocorticoid receptor antagonist; PA, primary aldosteronism; LRH, low-renin essential hypertension; W, weeks; M, months; Y, year. Changes of target renin rates in all participants (**A**, n=163), PA patients (**B**, n=123), and LRH patients (**C**, n=40) during follow-up after initiation of MRA. Patients who reached target renin are shown in black bars, those who continued to have suppressed renin in white bars, and those in whom renin was not assessed in grey bars.

Patients were followed for a median of 124 (interquartile range: 65, 335) days while on MRA treatment. In 59% of cases (74/123 with PA and 22/40 with LRH), the MRA titration decision was based on renin values or side effects. In a minority of patients, MRA titration was based on other factors, such as blood pressure (14%, 11 with PA and 12 with LRH)) and serum potassium (4%, 3 with PA and 1 with LRH), or no documentation regarding the rationale for the titration was found (9%, 10 with PA and 4 with LRH). In the remaining patients, the initial MRA doses were not changed. The median MRA dose used at the last visit was 50 (range, 12.5 to 300) mg/day (**Table 1**). Most patients (82%) were treated with spironolactone. The overall cumulative proportion of patients in whom target renin was achieved was 43% at the last visit (**Figure 2A**). The proportion of patients with target renin increased gradually over time in both groups, but was higher across all stages in patients with LRH as compared to the PA group. In the PA group, target renin was achieved in 2% of patients at 2 weeks, and it reached 40% by the last visit (**Figure 2B**). In patients with LRH, target renin rates were reached in 8% at 2 weeks and 53% one year after MRA initiation (**Figure 2C**).

### Factors Associated With Target Renin in PA and LRH

The clinical characteristics of PA patients with and without achievement of target renin levels are summarized in **Table 2A**. Compared to those who achieved target renin levels, patients with persistent renin suppression were younger (54 [44, 62] vs. 59 [51, 67] years, *p*=0.01), were more often treated with beta-blockers (74% vs. 51%, *p*=0.008), and had lower serum potassium concentrations (3.7 vs 3.9 mM, *p*=0.001). Patients with PA who reached a target renin value at the last visit were treated with higher doses of MRAs (50.0 [25.0, 81.3] vs. 31.3 [25.0, 50.0] mg/day, *p*=0.002), and had higher serum potassium levels (4.4 [4.2, 4.7] vs. 4.2 [3.9, 4.5] mM, *p*=0.01). Of the concomitant antihypertensive agents used, beta-blockers were associated with higher odds of persistent renin suppression (*p*=0.02), whereas neither RAAS inhibitors, nor potassium-wasting diuretics had an impact on the likelihood of target renin achievement. Multiple logistic regression determined that a lower baseline serum potassium, lower MRA dose, and beta-blocker use were independently associated with lower odds of achieving target renin levels (**Table 3A**).

**Table 2:** Comparison of patients with and without target renin following MRA therapy.

Variables		Target renin	Suppressed renin	*p*
**A. PA patients**				
N		49	74	
Age (years)		59 [51, 67]	54 [44, 62]	0.01
Women (N, %)		22 (44.9%)	27 (36.5%)	0.35
BMI (kg/m^2^)		33.6 [29.9, 36.8]	32.7 [28.3, 35.9]	0.35
SBP (mmHg)		155 [137, 172]	143 [126, 156]	0.01
DBP (mmHg)		84 [78, 90]	82 [72, 92]	0.41
Serum potassium (mM)		3.9 [3.6, 4.3]	3.7 [3.4, 4.0]	0.001
eGFR (mL/min/1.73m^2^)		84 [68, 96]	80 [63, 90]	0.38
PAC (ng/dL)		19.4 [15.9, 27.3]	22.0 [16.4, 30.8]	0.30
PRA (ng/mL/hr) [N=99]		0.3 [0.1, 0.6]	0.2 [0.1, 0.6]	0.33
DRC (pg/mL) [N=24]		2.1 [2.1, 5.1]	2.1 [2.1, 2.1]	0.50
ARR (ng/dL per ng/mL/h)		66.3 [38.3, 113.5]	100.2 [41.0, 210.0]	0.10
Confirmed unilateral PA cases (N, %)		5 (10.2%)	20 (27.0%)	0.02
Antihypertensive agents (N)		3.0 [1.0, 4.0]	3.0 [2.0, 4.0]	0.75
Beta-blocker (N, %)		25 (51.0%)	55 (74.3%)	0.008
Potassium replacement (N, %)		21 (42.9%)	34 (45.9%)	0.74
Cardiovascular disease (N, %)		8 (16.3%)	15 (20.3%)	0.58
Follow-up duration (days)		182 [78, 342]	164 [66, 342]	0.79
MRA dosage at the last visit (mg/day)		50.0 [25.0, 81.3]	31.3 [25.0, 50.0]	0.002
MRA-related side effect (N, %)		16 (32.7%)	25 (33.8%)	0.64
Serum potassium at the last visit (mM)		4.4 [4.2, 4.7]	4.2 [3.9, 4.5]	0.01
eGFR at the last visit (mL/min/1.73m^2^)		66 [56, 80]	72 [60, 91]	0.08
PAC at the last visit (ng/dL) [N=82]		32.1 [23.5, 48.0]	28.7 [20.4, 38.6]	0.14
PRA at the last visit (ng/mL/h) [N=92]		2.3 [1.3, 5.9]	0.3 [0.2, 0.6]	<0.001
DRC at the last visit (pg/mL) [N=14]		15.0 [12.9, 26.5]	2.5 [2.1, 4.4]	<0.001
ARR at the last visit (ng/dL per ng/mL/h) [N=82]		15.4 [8.7, 20.4]	76.0 [44.3, 128.1]	<0.001
**B. LRH patients**				
N		21	19	
Age (years)		47 [36, 68]	59 [50, 64]	0.24
Women (N, %)		10 (47.6%)	17 (89.5%)	0.005
BMI (kg/m^2^)		35.4 [29.7, 40.9]	30.2 [26.6, 35.6]	0.08
SBP (mmHg)		157 [143, 174]	160 [130, 184]	0.50
DBP (mmHg)		87 [80, 98]	83 [72, 106]	0.84
Serum potassium (mM)		4.0 [3.5, 4.2]	3.9 [3.5, 4.3]	0.71
eGFR (mL/min/1.73m^2^)		77 [58, 95]	87 [75, 103]	0.26
PAC (ng/dL)		11.0 [8.6, 16.4]	11.3 [8.3, 17.3]	0.94
PRA (ng/mL/hr) [N=23]		0.6 [0.3, 0.7]	0.2 [0.1, 0.4]	0.04
DRC (pg/mL) [N=17]		2.7 [2.1, 6.6]	2.3 [2.1, 3.9]	0.79
ARR (ng/dL per ng/mL/h)		25.8 [13.6, 38.4]	36.1 [25.5, 58.8]	0.11
Antihypertensive agents (N)		3.0 [1.8, 4.0]	3.0 [2.0, 3.8]	0.72
Beta-blocker (N, %)		8 (38.1%)	7 (36.8%)	0.93
Potassium replacement (N, %)		4 (19.0%)	4 (21.1%)	0.87
Cardiovascular disease (N, %)		6 (28.6%)	4 (21.1%)	0.58
Follow-up duration (days)		80 [31, 160]	76 [37, 184]	0.99
MRA dosage at the last visit (mg/day)		25.0 [25.0, 50.0]	25.0 [25.0, 50.0]	0.84
MRA-related side effect (N, %)		6 (28.6%)	3 (15.8%)	0.33
Serum potassium at the last visit (mM)		4.5 [4.2, 4.9]	4.2 [4.0, 4.4]	0.02
eGFR at the last visit (mL/min/1.73m^2^)		65 [43, 94]	75 [60, 82]	0.25
PAC at the last visit (ng/dL) [N=82]		19.0 [14.3, 26.5]	13.4 [8.5, 18.5]	0.02
PRA at the last visit (ng/mL/h) [N=92]		2.6 [1.5, 5.4]	0.3 [0.2, 0.5]	<0.001
DRC at the last visit (pg/mL) [N=14]		19.7 [18.4, 77.0]	3.1 [2.2, 3.4]	<0.001
ARR at the last visit (ng/dL per ng/mL/h) [N=82]		5.9 [1.9, 17.4]	47.0 [25.7, 54.0]	<0.001

PA, primary aldosteronism; LRH, low renin essential hypertension; BMI, body mass index; SBP, systolic blood pressure; DBP, diastolic blood pressure; eGFR, estimated glomerular filtration ratio; PAC, plasma aldosterone concentration; PRA, plasma renin activity; DRC, direct renin concentration; ARR, aldosterone-to-renin ratio; MRA, mineralocorticoid receptor antagonist. Continuous variables are shown as median [interquartile range].

**Table 3:** Factors associated with target renin achievement during MRA therapy.

	β	SE	*p*	Odds ratio	95% CI
**A. PA patients**					
Serum potassium at baseline (mM)	1.67	0.48	<0.001	5.32	2.07-16.68
Beta-blocker use (Reference: no use)	-1.29	0.44	0.004	0.28	0.12-0.66
MRA dose (mg/day)	0.02	0.0056	0.002	1.018	1.01-1.03
**B. LRH patients**					
Sex (Reference: women)	2.24	0.87	0.01	9.35	1.71-51.03

PA, primary aldosteronism; LRH, low renin essential hypertension; MRA, mineralocorticoid receptor antagonist; SE, standard error; CI, confidence interval. Serum potassium, beta-blocker use and MRA daily dosage in PA and sex in LRH were chosen for multiple logistic regression analysis, using a backward stepwise selection.

Of patients with LRH, those who reached target renin levels had higher PRA (0.6 [0.3, 0.7] vs. 0.2 [0.1, 0.4] ng/mL/h, *p*=0.04) at baseline than the patients with persistent renin suppression (**Table 2B**). Intriguingly, patients with persistent renin suppression were more frequently women than those with target renin achievement (90% vs. 48%, *p*=0.005). There was no difference in age, beta-blocker use, or MRA doses between the two groups. In multiple logistic regression, only sex was independently associated with target renin levels (**Table 3B**).

### Safety and Side Effects of MRA Therapy

Overall, serum potassium concentrations increased (from 3.8 [3.5, 4.2] to 4.3 [4.1, 4.6] mM, *p*<0.001 for all) during MRA treatment. The percentage of patients taking potassium replacement therapy decreased from 39% to 23% (*p*<0.001), and 21 (13%) patients developed hyperkalemia (**Supplemental Table**). Of the latter, 16 (76%) patients had chronic kidney disease (CKD), and 12 (57%) were concurrently treated with RAAS inhibitors. Overall, eGFR decreased, from 81 [64, 94] prior to MRA initiation to 69 [57, 83] mL/min/1.73m^2^ (*p*<0.001) at the last follow-up visit. The changes in eGFR and renin were inversely associated in PA (*r*=-0.3022, *p*=0.002, **Supplemental Figure 2**), but not in LRH patients (data not shown).

Gynecomastia and/or breast tenderness occurred in 21 (13%) patients taking spironolactone, at doses between 25-200 mg daily (**Supplemental Table**). Other side effects associated with spironolactone included: acute elevation of creatinine, irregular vaginal bleeding, and decreased libido (**Supplemental Table**). The incidence of side effects was similar between patients with and without PA (33% vs. 23%, *p*=0.20). Overall, side effects occurred after a median follow up of 91 days and while taking MRA doses between 12.5-400 mg/day. In 17 patients (34%), spironolactone was changed to eplerenone, and 9 other patients (18%) stopped or reduced the doses of spironolactone.

## Discussion

Although MRAs have been used primarily for blood pressure control in patients with PA and resistant hypertension (31), recent data indicate that the risk of cardio-renal complications is reduced only in patients with adequate MR blockade, as suggested by reversal of renin suppression (17, 18). Several noteworthy findings emerge from this study of clinical practice patterns in an academic center, with a large volume of patients with hypertension: 1) a very small fraction of patients with hypertension are treated with MRAs; 2) in very few patients treated with MRAs the dose is titrated based on renin targets; 3) only 43% of patients with PA and other LRH treated with MRAs reached target renin after one year of treatment. These findings suggest that a large proportion of patients with hypertension are exposed to preventable risk of cardiovascular and renal morbidity and even death.

While patients with unilateral PA can be cured with surgery, patients with bilateral PA or those who do not undergo unilateral adrenalectomy require life-long medical management. In the absence of selective aldosterone synthase inhibitors, MRAs are the first line of treatment for all non-surgical PA cases (13). Evidence from cohort studies of patients with PA and essential hypertension suggests that, overall, PA patients treated with MRAs have an excess risk of developing atrial fibrillation and renal insufficiency as compared to those treated surgically or patients with essential hypertension with similar baseline blood pressure and risk factors (4, 18). The excessive risk of cardiovascular events and even mortality appeared to be annulled in patients treated with MRA doses that allowed renin elevations above 1 μg/L/h (17). MRAs are also highly effective add-on therapies for controlling resistant hypertension cases (19, 25), likely due to the high prevalence of unrecognized PA and other LRH (3). Moreover, mounting evidence suggests that PA spans a hormonal and clinical continuum, and that some cases of LRH might represent early stages of PA (32, 33). Yet, MRAs are prescribed infrequently. In line with previous data (34, 35), only 5% of all hypertensive patients and less than 10% of those diagnosed with hyperaldosteronism were prescribed MRAs in our cohort. Furthermore, of those who received MRAs, renin goals were followed in a minority of patients and were achieved in under half of the latter subgroup.

In addition to the lack of awareness regarding the benefits of MRA treatment in patients with low-renin hypertension, concerns about hyperkalemia might limit their broader use. The risk of hyperkalemia increases in patients with renal insufficiency. PA is associated with intravascular volume expansion and glomerular hyperfiltration. In such patients, correction of the hyperaldosteronism, either by surgery or medical treatment with MRA, can unmask underlying CKD, by reinstalling normal intravascular volume states (36, 37). Adrenalectomy for aldosterone-producing adenomas leads to a decline in eGFR by 11 to 15 mL/min/1.73m^2^, and increase the proportion of apparent CKD (37–39). Similarly, MRA therapy in bilateral PA might cause a relatively acute fall of eGFR, but contributes to long-term preservation of renal function (40, 41). The inverse association between changes of renin and eGFR observed in our study is in line with previous data. Taken together these results indicate that eGFR should be carefully monitored after MRA initiation, and that a mild decline in eGFR should not prompt MRA discontinuation, as cardio-protective benefits occur with long-term MRA therapy.

Simultaneously with the escalation of MRA doses, adjustments of other medications that influence eGFR and/or serum potassium concentrations are essential to prevent hyperkalemia while attempting to reverse renin suppression. In order to reduce the risk of hyperkalemia, discontinuation of potassium supplements and reduction of RAAS inhibitors must be anticipated when suppressed renin renders intensification of MRA therapy. In our study, 21 patient developed hyperkalemia, and of these, 57% were concomitantly taking angiotensin converting enzyme inhibitors or angiotensin receptor blockers, and 76% had CKD.

In addition to the impact of potassium and renal function, concomitant drugs that interfere with RAAS might alter the time to achieving goal renin levels (13, 42). In particular, beta-blockers decrease both renin activity and concentration by blocking sympathetic stimulation (42, 43). Supporting these effects, our study demonstrates that beta-blockers are associated with lower odds of overcoming renin suppression during MRA treatment. Beta-blockers are often used in patients with cardiac pathology, including heart failure, coronary artery disease, and atrial fibrillation or other tachyarrhythmias, and have survival benefits in such patients (44, 45). Thus, clinical trials designed to answer how to best manage patients with low renin hypertension and cardiac history are greatly needed.

In addition to concerns related to polypharmacy, medication-specific side effects can impact patients’ adherence to recommended treatments. In this study, 14% of patients experienced breast tenderness, breast enlargement and/or sexual dysfunction while taking spironolactone. Spironolactone was the first MRA developed and it has been commercialized since the 1950s. While spironolactone is a potent MRA, it can also block the androgen and progesterone receptors (46, 47), which explain its side effect profile. Eplerenone has superior MR selectivity and low propensity for gynecomastia (47, 48), but its efficacy is lower than that of spironolactone (27, 28). Nevertheless, prospective and retrospective studies have shown that both MRAs are efficacious for blood pressure control and reduction of morbidity and mortality associated with heart failure (49–51). Consequently, eplerenone might offer an advantage over spironolactone, particularly in men.

Our study has several limitations, including its retrospective design, heterogeneity of concomitant antihypertensive regimens, and variability in follow-up. In addition, due to the overwhelming number of hypertensive patients and limitation of our medical records search engine, it was not feasible to determine the exact number of patients with resistant hypertension. Nevertheless, extrapolating from existing data, we speculate that a large number of patients who could benefit from MRA are never prescribed these agents.

In summary, in this study of patients with hypertension seen in a large academic center, including primary care and hypertension-focused services, we found that MRAs are rarely prescribed. Despite compelling evidence that inappropriate MR activation enhances the risk of cardiorenal morbidity and mortality, MRA titration to doses that overcome MR activation and reverse renin suppression is pursued in a minority of patients with PA and LRH, and is achieved in less than half of those followed. Until large prospective longitudinal studies of patients with PA and other low-renin hypertension will elucidate the optimal parameters for guiding medical therapy titration, incorporating renin targets within PA practice guidelines is likely to benefit patient care.

## Data Availability Statement

The datasets presented in this article are not readily available due to patient privacy. Requests to access the datasets should be directed to aturcu@umich.edu.

## Ethics Statement

The studies involving human participants were reviewed and approved by University of Michigan IRB. Written informed consent for participation was not required for this study in accordance with the national legislation and the institutional requirements.

## Author Contributions

AFT conceptualized the study. AFT and YT designed the study. YT collected the data and performed the statistical analysis. AFT was responsible for reviewing the data and providing scientific input. YT and AFT wrote the article. All authors contributed to the article and approved the submitted version.

## Funding

AFT was supported by grants: 1K08DK109116 from the NIDDK, 2019087 from the Doris Duke Charitable Foundation, and U070002 from the Michigan Institute for Clinical & Health Research (MICHR).

## Conflict of Interest

The authors declare that the research was conducted in the absence of any commercial or financial relationships that could be construed as a potential conflict of interest.

## Abbreviations

CKD, chronic kidney disease; DRC, direct renin concentration; eGFR, estimated glomerular filtration ratio; LRH, low renin essential hypertension; MR, mineralocorticoid receptor; MRA, mineralocorticoid receptor antagonist; PA, primary aldosteronism; PAC, plasma aldosterone concentration; PRA, plasma renin activity; RAAS, renin-angiotensin-aldosterone system.
