# Supplementary material for: Real-World Effectiveness of Mineralocorticoid Receptor Antagonists in Primary Aldosteronism
Source: Front Endocrinol (Lausanne). 2021 Mar 26;12:625457. doi: 10.3389/fendo.2021.625457 (PMC8033169; doi:10.3389/fendo.2021.625457)
Supplement: Supplementary file 1 [file DataSheet_1.docx]

**Supplemental Table**. Common side effects associated with MRA use

| **Side effect** | **N** | **Spironolactone dose (mg/day)** | **Treatment duration (days)** |
| --- | --- | --- | --- |
| Gynecomastia | 21 | 25-200 | 3-321 |
| Hyperkalemia | 21 | 25-400 | 9-371 |
| Acute creatinine elevation | 2 | 50 | 65-77 |
| Other symptoms^a^ | 11 | 12.5-50 | 2-371 |

MRA, mineralocorticoid receptor antagonist. The doses of spironolactone and treatment period are shown as ranges.

^a^ Other reported symptoms included: irregular menses, decreased libido, rash, fatigue, lightheadedness, angioedema, and frequent urination.

**Supplemental figure 1**. Numbers of patients per years studied

PA, primary aldosteronism.


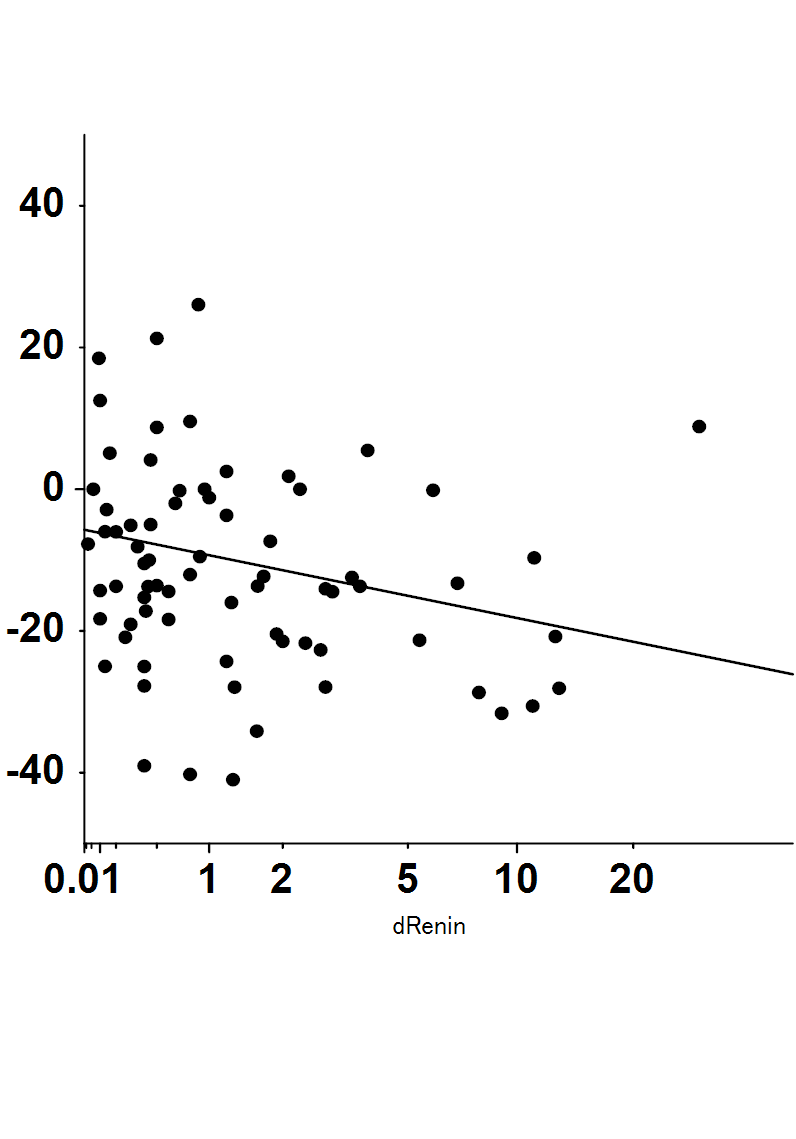


𝜟 eGFR

*r*=-0.302, *p*=.002

mL/min/1.73m^2^

ng/mL/h

𝜟 PRA

**Supplemental Figure 2**. Correlations between renin and eGFR changes in patients with PA treated with MRAs

PA, primary aldosteronism; eGFR, estimated glomerular filtration ratio; PRA, plasma renin activity; DRC, direct renin concentration; MRA, mineralocorticoid receptor antagonist.

DRC was converted to PRA using a coefficient factor of DRC/PRA of 8.
